# Maternal and community factors associated with unmet contraceptive need among childbearing women in Northern Nigeria

**DOI:** 10.1186/s40834-019-0093-1

**Published:** 2019-09-02

**Authors:** Bola Lukman Solanke, Funmilola Folasade Oyinlola, Olaoye James Oyeleye, Benjamin Bukky Ilesanmi

**Affiliations:** 1 Department of Demography and Social Statistics, Obafemi Awolowo University, Ile-Ife, Nigeria; 2 Action Against Hunger, ACF-International, Damaturu, Yobe State Nigeria

**Keywords:** Unmet need, Contraceptive, Reproductive health, Women, Maternal health, Northern Nigeria

## Abstract

**Background:**

Unmet need for modern contraceptive remains a critical reproductive health challenge in Nigeria. Numerous studies in Nigeria and other countries have investigated the patterns, prevalence and associated factors of unmet contraceptive need. In spite of these, the associated factors of unmet contraceptive need in Northern Nigeria have remained insufficiently explored. The few studies that focused on Northern Nigeria have mainly examined maternal individual factors leaving out higher level factors such as community-level factors that may be associated with unmet contraceptive need. This study examines the extent to which maternal and community factors are associated with unmet contraceptive need in Northern Nigeria.

**Method:**

Data was pooled from 2008 to 2013 Nigeria Demographic and Health Surveys. A weighted sample size of 26,730 women was analysed. The outcome variable was unmet contraceptive need, dichotomised into no unmet need and unmet need. The explanatory variables were individual maternal characteristics such as age, education, number of living children, age at marriage, pregnancy termination experience, and death of a child, and selected community characteristics such as community socioeconomic status, community literacy level, community knowledge of modern contraceptive and geo-political zone. The Multilevel Logistic Regression Model (MLRM) was applied.

**Result:**

Results showed a prevalence of 18% unmet contraceptive need among Northern women in Nigeria. Maternal age of 35 years or older (AOR = 0.873; *p* < 0.05, CI: 0.780–0.976), having five or more living children (AOR = 1.813; *p* < 0.001, CI: 1.663–1.977), higher maternal education (AOR = 0.787; *p* < 0.05, CI: 0.625–0.993), and never experience death of a child (AOR = 0.866; *p* < 0.001, CI: 0.805–0.933) are the maternal factors significantly associated with unmet contraceptive need, while high community literacy level (AOR = 1.230; *p* < 0.05, CI: 1.041–1.454), moderate (AOR = 0.862; *p* < 0.05, CI: 0.767–0.968) or high (AOR = 0.821; *p* < 0.05, CI: 0.726–0.929) community knowledge of modern contraceptive, and geo-political zone of residence are the community-level characteristics significantly associated with unmet contraceptive need among women in Northern Nigeria.

**Conclusion:**

Maternal and community factors are significantly associated with unmet contraceptive need, but based on the ICC maternal factors have more significance in Northern Nigeria. The expansion of existing family planning delivery points to cover all communities including rural and remote areas in the region is imperative.

## Background

Unmet contraceptive need exists when women desire to limit childbearing or delay next pregnancy, but are not using any form of contraception to actualise such reproductive desire [1–3]. Unmet contraceptive need remains a public health concern in developing countries particularly in sub-Saharan Africa, where it is currently high but expected to reduce if the countries increase public expenditure on family planning and improve access to modern contraceptives [4, 5]. It is important to address the current high unmet contraceptive need in developing countries because it elevates the exposure of childbearing women to unintended pregnancies, high-risk births, and unsafe abortion, in addition to preventing women from engaging in optimal economic productivity, and thus, may undermine the attainment of the post-2015 development agenda [6–9].

Numerous studies in Nigeria [10–12] and in other countries [13–19] have investigated the patterns, prevalence and associated factors of unmet contraceptive need. These studies not only provided information on the various reasons for unmet contraceptive need among women, but also raise public awareness on the consequences of unmet contraceptive need. In spite of these numerous studies, the associated factors of unmet contraceptive need in Northern Nigeria have remained insufficiently explored. The few studies that focused on Northern Nigeria [20–22] have mainly examined maternal individual factors associated with unmet contraceptive need. The studies provided evidence of low use of modern contraceptive among women in Northern Nigeria. A particular study found 10.3% unmet need for family planning among rural women in the region [21], which was much higher than the 2% reported in a more recent study in the region [22]. The studies also provided evidence of male control over women’s utilisation of modern contraceptives in the region [21, 22]. Higher levels of influence on unmet contraceptive need such as community-level characteristics were largely ignored in the existing Northern Nigerian studies.

As observed in an earlier study [23], the determinants of low use of modern contraceptives, and high unmet need among women in low and middle-income countries are similar and operate at multiple levels of the social and physical environment such as the individual level, household level, community level, and health service level. Each level of influence has both joint and independent effects on the level of contraceptive use as well as level of unmet contraceptive need in specific context. For instance, two women with similar or the same socio-demographic characteristics may have different likelihood of contraceptive use due to living in different communities with different proportions of women who had economic empowerment in the community. By examining factors beyond the maternal level, the research prospect of identifying more relevant factors to be targeted in family planning interventions is enhanced across developing countries. It is against this backdrop that the current study focused on maternal and community factors associated with unmet contraceptive need in Northern Nigeria. The study is guided by the research question: to what extent are maternal and community factors associated with unmet contraceptive need in Northern Nigeria?

## Methods

### Study context

The Federal Republic of Nigeria, an Anglo-phone West African country is the current seventh most populous country in the world, and Africa’s most populous country [24]. Population within the country is unevenly distributed between the Southern and Northern regions of the country, with the Northern region being the most populous region in the country. However, in terms of socio-economic development, the Northern region is the poorest in the country with several parts of the region currently undergoing armed insurgency that have gone a long way in reducing availability and utilisation of reproductive health services including family planning services [25–27]. The Northern region of Nigeria is predominantly Islamic with well-entrenched patriarchal social order that thrives on polygynous marriages which has been associated with lower contraceptive use in the country [28]. The region is a high fertility zone [29] with persistence of high parity [30, 31] which has been associated with adverse maternal and child health outcomes in Northern Nigeria [32, 33], as well as in other countries [34–36], and low contraceptive use among high parous women [37]. The region also had the least percentage of family planning demand satisfied by modern methods in the country [38]. Nevertheless, many women in the region still face fertility challenges [39]. Most other indicators of demographic and health situation in the country such as contraceptive prevalence, infant and child health, maternal mortality, and female genital mutilation/cutting reveal that the Northern region is not only worst off in the country, but also deserves more reproductive health attention, which is been promoted through series of health initiatives [40] in addition to ongoing implementation of national population and health policies [41–43].

### Data source and sample size

Data analysed in the study was pooled from 2008 and 2013 Nigeria Demographic and Health Surveys (NDHSs) which was part of the Demographic and Health Survey (DHS) being implemented across developing countries to provide national estimates of basic demographic and health information such as fertility, family planning, maternal and child health, female genital mutilation/cutting, and domestic violence. The DHS are nationally representative surveys based on similar methodology and design across the developing countries [44]. In each country where the surveys are conducted, the national population or statistical agency is usually the agency to implement the survey with technical and financial support from the USAID through MEASURE DHS. The 2008 and 2013 NDHSs are the fourth and fifth rounds of the survey in Nigeria. Samples in the surveys were selected in a three-staged stratified cluster designs. In both surveys, information was obtained from respondents using DHS model questionnaires. Interviews were conducted sequel to obtaining informed consent from respondents. The design of the surveys has been widely published [38, 45]. In the current study, the 2008–2013 data were pooled to form a large mass of data that enhance statistical precision and inference. The study used the survey weights provided by the DHS to weight the sample. Hence, a weighted sample size of 26,730 women was analysed. Women included in the sample were: women who want to postpone their next pregnancy or birth but not using a contraceptive method; women who want no more children but not using a contraceptive method; and women who either had a current mistimed or unwanted pregnancy. Those excluded from the study were women from the Southern region and other women that are not relevant to the study such as women who are not currently married, and those not sexually active.

### Research variables

The outcome variable was unmet contraceptive need, which was categorised into unmet need and no unmet need. The category of interest in the study was the unmet need category. This group represents the proportion of women who desire to either delay the next pregnancy or limit child birth, but not using any method of contraception [1–3]. The explanatory variables were individual maternal characteristics such as age, age at marriage, number of living children, pregnancy termination experience, education, female autonomy on household decision, and experience of death of a child, and community-level characteristics such as community socioeconomic status, community literacy level, community knowledge of modern contraceptive, proportion of women ever used modern contraceptive in community, place of residence, and geo-political zone. These variables were selected based on their significance in previous studies [11, 12, 14, 15, 23]. Four community-level characteristics, namely, community education level, community socioeconomic status, proportion ever used modern contraceptive method, and community knowledge of modern contraceptive were divided into three categories of ‘low, medium, and high’ based on the percentiles. Two health service factors, namely, visitation by family planning worker and barriers to healthcare, and two male-partner factors, namely partner education and couple fertility desire were selected for statistical control in the study. Table 1 provides further information on variable definition and measurement.

**Table 1:** Variable definition and measurement

S/No.	Name of variable	Measurement	Category/code
Outcome variable			
1.	Unmet contraceptive need	Women who desire to delay next pregnancy or limit child birth, but not using any form of contraception	1. Unmet contraceptive need 0. No unmet need
Individual maternal characteristics			
2.	Age	Current age of respondent	0. 15–24 years 1. 25–34 years 2. 35 years or older
3.	Education	Maternal level of educational attainment	1. None 2. Primary 3. Secondary 4. Higher
4.	Age at first marriage	Age of respondent at the first marriage	1. 17 years or less 2. 18–24 years 3. 25 years or older
5.	Female autonomy	The sole, joint or non-participation of women in three household decision of own healthcare, purchase of large items, and visit to friends and family	1. Full autonomy 2. Joint autonomy 3. No autonomy
6.	Pregnancy termination	Respondents’ experience of pregnancy termination	1. Never experienced 2. Ever experienced
7.	Child mortality experience	Whether respondent has experience or never experienced death of a child	1. Never experienced 2. Ever experienced
8.	Number of living children	Respondent number of children ever born that are alive	1. Four or less 2. Five or more
Partner and health service factors (Control variables)			
9.	Partner education	Educational attainment of respondent male partner	1. None 2. Primary 3. Secondary 4. Higher
10.	Couple fertility desire	The fertility preference of respondent and the male partner on family size	1. Both want same 2. Husband want more 3. Husband want fewer 4. Don’t know
11.	Visitation by family planning worker	Whether respondent was visited by a family planning worker in the last 12 months preceding the survey	1. Not visited 2. Visited
12.	Barriers to healthcare	Whether respondent perceived that getting partner permission to access healthcare, getting money for treatment, or distance to health facility constitute a barrier to accessing healthcare	1. No barrier 2. At least one barrier
Community characteristics (Higher level of influence)			
13.	Community education level	The proportion of women who had at least secondary education in the community. Not directly available in data but generated from maternal education through method of aggregation at the cluster level	1. Low 2. Medium 3. High
14.	Community socioeconomic status	The proportion of women in richest household wealth quintile in the community. Not directly available in data but generated from household wealth quintile through method of aggregation at the cluster level	1. Low 2. Medium 3. High
15.	Proportion ever used modern contraceptive in community	The extent to which women in the community had ever used modern contraceptive method. Not available directly in data but generated from women’s ever use of modern contraceptive method through method of aggregation at the cluster level	1. Low 2. Medium 3. High
16.	Community knowledge of modern contraceptive	The proportion of women in the community who have knowledge of at least one modern contraceptive. Not directly available in data but generated through method of aggregation at the cluster level	1. Low 2. Medium 3. High
17.	Place of residence	Whether respondent place of residence is rural or urban type community	1. Urban 2. Rural
18.	Geo-political zone	The geographic/administrative residence of sampled women	1. North-central 2. North-east 3. North-west

### Data analysis

Data were analysed at the univariate, bivariate and multivariate levels. Prevalence of unmet contraceptive need was described using the pie chart, while the respondents’ characteristics were described using percentages and frequency distribution at the univariate level. At the bivariate level, Unadjusted Odds Ratio (UOR) of binary logistic regression was used to examine the likelihood of unmet contraceptive need due to change in each specific explanatory variable. Variables that show statistical significance at this level were selected for inclusion in Multilevel Logistic Regression Model (MLRM). The MLRM was used based on the hierarchy of influence on unmet contraceptive need, namely, individual maternal level and community level, and to show effects that vary by community. The MLRM consists of fixed effects (regression coefficients that vary but not being modelled), and random effects (the regression coefficients being modelled) [46, 47].

The MLRM fitted in the study was specified as:

$$ logit\ \left({P}_r\left({Y}_{ij}=1\right)\right)={\alpha}_0+{\alpha}_{oj}+{\alpha}_1{x}_{1 ij}+\dots +{\alpha}_k{x}_{kij}+{\beta}_1{z}_{1j}+\dots {\beta}_m{z}_{mj} $$

Where:

*x*_*ij*_ − *x*_*kij*_ are the maternal characteristics

*z*_1*j*_ − *z*_*mj*_ are the community-level characteristics

The fixed effects of MLRM were assessed using the binary logistic Adjusted Odds Ratio (AOR), while the random effects of the MLRM were measured using Intra-Cluster Correlation (ICC) and Median Odds Ratio (MOR). The ICC calculated as:$\frac{\sigma_{ui}^2}{\sigma_{ui}^2+\left[\frac{\pi^2}{3}\right]}$ where ${\sigma}_{ui}^2$ is the variance at the community level [48] shows the variation in unmet contraceptive need due to the community-level characteristics, and ranges from 0 to 1 but may be expressed in percentage. The higher the values of the ICC, the more important are the community-level characteristics. The MOR calculated as: $\exp (0.95)\sqrt{\sigma_{ui}^2}$ [49] is the median increased odds of reporting unmet contraceptive need if a woman moves to another community with higher likelihood of unmet contraceptive need. The higher the MOR, the more are the importance of the community-level characteristics in predicting variations in unmet contraceptive need. In the absence of community-level characteristics, the value of MOR will be equal to 1 [48]. Model adequacy was checked using the LR test. Statistical significance is accepted at *p* < 0.05. All analyses were performed using Stata version 14 [50].

## Results

**Fig. 1:**
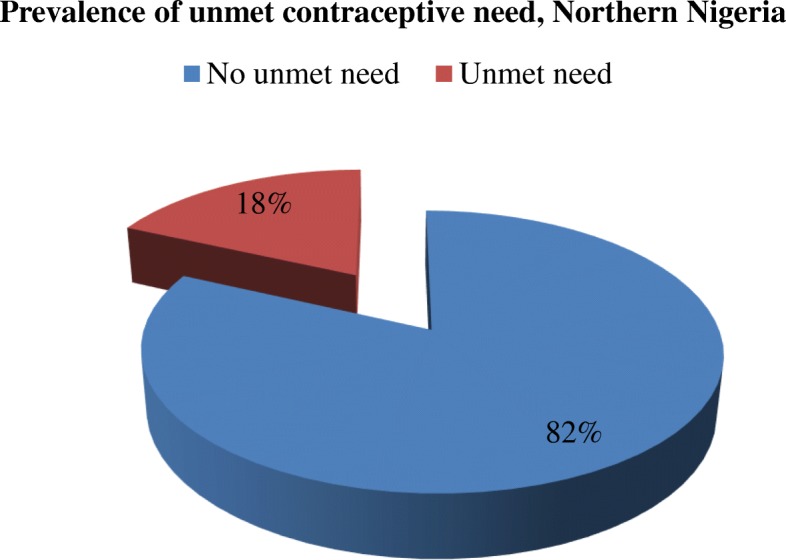
Prevalence of unmet contraceptive need in Northern Nigeria

Figure 1 presents the prevalence of unmet contraceptive need among the respondents. As shown in the figure, nearly one-fifth of the respondents had unmet contraceptive need, while the majority of the respondents had no unmet contraceptive need. Table 2 presents the socio-demographic characteristics of the respondents. Slightly more than a quarter of the respondents were less than 25 years, while the rest were 25 years or older. The majority of respondents were 17 years or younger at the time of marriage, while slightly more than one-fifth of the respondents were in the age range of 18 to 24 years at the time of their marriage. The majority of the respondents had four or fewer number of living children, while less than one-third of the respondents had five or more living children. The majority of the respondents had no formal education, but the dominant educational attainment among the educated was primary education. The majority of respondents had no autonomy on household decisions. Slightly more than one-tenth of the respondents had experienced at least one pregnancy termination, while more than half of the women had never experienced the death of a child.

**Table 2:** Respondents’ Socio-demographic characteristics

Characteristic	Number of Women (*n* = 26,730)	Percentage
Maternal age		
15–24 years	7870	29.4
25–34 years	9785	36.6
35 years or older	9075	34.0
Age at first marriage		
17 years or younger	20,192	75.5
18–24 years	5619	21.0
25 years or older	919	3.5
Number of living children		
Four or less	18,775	70.2
Five or more	7955	29.8
Education		
None	18,851	70.5
Primary	3990	14.9
Secondary	3076	11.5
Higher	813	3.1
Female autonomy		
Sole	220	0.8
Joint	4631	17.3
No autonomy	21,879	81.9
Pregnancy termination		
Never experienced	23,432	87.7
Ever experienced	3298	12.3
Death of a child		
Ever experienced	10,818	40.5
Never experienced	15,912	59.5
Partner education		
None	15,864	59.4
Primary	3840	14.4
Secondary	4422	16.5
Higher	2604	9.7
Barriers to healthcare		
At least one barrier	3220	12.1
No barrier	23,510	87.9
Visitation by family planning worker		
Not visited	25,813	96.6
Visited	917	3.4
Couple fertility desire		
Both want same	6526	24.4
Husband want more	11,874	44.4
Husband want fewer	859	3.2
Don’t know	7471	28.0
Place of residence		
Urban	6104	22.8
Rural	20,626	77.2
Community literacy level		
Low	9476	35.4
Medium	8916	33.4
High	8338	31.2
Community socioeconomic status		
Low	12,174	45.5
Medium	5642	21.1
High	8914	33.4
Community knowledge of modern contraceptive		
Low	8741	32.7
Medium	8840	33.1
High	9149	34.2
Proportion ever used contraceptive in community		
Low	9648	36.1
Medium	9058	33.9
High	8023	30.0
Geo-political zone		
North-central	4680	17.5
North-east	6898	25.8
North-west	15,152	56.7

*Source:* Author analysis based on 2008–2013 NDHSs

The distribution of the respondents by community literacy level was nearly equal among the respondents. However, slightly higher proportion of the respondents lives in communities with low proportion of women who had secondary education compared to other community educational categories. Nearly half of the respondents live in communities with low socioeconomic status, while one-third of the respondents live in communities with high socioeconomic status. The distribution of respondents by community knowledge of modern contraceptive was nearly equal among the women. But higher proportions of the respondents live in communities with low proportion of women who had ever used contraceptive. The majority of respondents are rural dwellers. Respondents from the North-west geo-political zone were dominant in the sample. Virtually all the respondents were not visited by a family planning worker in the last 12 months preceding the survey. The majority of respondents had no barrier to accessing healthcare. More than half of respondents’ partners had no formal education, but secondary education was the dominant level among educated partners. More than two-fifths of respondents’ partners desired more children compared to the respondents.

Table 3 presents the bivariate results of the analyses. As maternal age increases, the likelihood of unmet contraceptive need increased significantly among the women particularly among women in the advanced reproductive age of 35 years or older (UOR = 1.335; *p* < 0.001). Likewise, as number of living children increased from four or less to five or more, the likelihood of unmet contraceptive need increased significantly among the women (UOR = 1.672; *p* < 0.001). On the contrary, as age at marriage increases, the likelihood of unmet contraceptive need reduced among the respondent, but this was without statistical significance. Maternal education had mixed relationship with unmet contraceptive need. At the primary and secondary educational levels, the likelihood of unmet need increased, but at higher educational level, the likelihood of unmet contraceptive need reduced significantly (UOR = 0.817; *p* < 0.05). Female autonomy and pregnancy termination were not significantly related to unmet contraceptive need among the women. Women who had never experienced death of a child were less likely to have unmet contraceptive need compared with women who had experienced death of a child (UOR = 0.832; *p* < 0.001).

**Table 3:** Unadjusted odds ratio of binary logistic regression showing association between specific explanatory variable and unmet contraceptive need

Variable	UOR	*p*-value	95% CI
Maternal age			
15–24 years ^a^	–	–	–
25–34 years	1.162*	*p* < 0.001	1.074–1.258
35 years or older	1.335*	*p* < 0.001	1.234–1.444
Age at first marriage			
17 years or younger^a^	–	–	–
18–24 years	0.997	0.933	0.925–1.074
25 years or older	0.915	0.278	0.779–1.075
Number of living children			
Four or less ^a^	–	–	–
Five or more	1.672*	*p* < 0.001	1.568–1.782
Maternal education			
None ^a^	–	–	–
Primary	1.102**	*p* < 0.05	1.013–1.199
Secondary	1.072	0.152	0.975–1.179
Higher	0.817	*p* < 0.05	0.686–0.972
Female autonomy			
Sole	–	–	–
Joint	0.991	0.955	0.715–1.373
No autonomy	0.913	0.579	0.663–1.258
Pregnancy termination			
Never experienced ^a^	–	–	–
Ever experienced	1.089	0.065	0.995–1.192
Death of a child			
Ever experienced ^a^	–	–	–
Never experienced	0.832*	*p* < 0.001	0.782–0.885
Partner education			
None ^a^	–	–	–
Primary	1.038	0.419	0.949–1.135
Secondary	1.109**	*p* < 0.05	1.019–1.206
Higher	1.038	0.468	0.938–1.149
Barriers to healthcare			
At least one barrier ^a^	–	–	–
No barrier	1.191**	0.001	1.077–1.318
Visitation by family planning worker			
Not visited ^a^	–	–	–
Visited	0.888	0.171	0.750–1.052
Couple fertility desire			
Both want same ^a^	–	–	–
Husband want more	1.004	0.911	0.929–1.085
Husband want fewer	1.025	0.790	0.855–1.229
Don’t know	1.180*	*p* < 0.001	1.085–1.283
Place of residence			
Urban ^a^	–	–	–
Rural	0.901**	0.005	0.838–0.969
Community literacy level			
Low ^a^	–	–	–
Medium	1.091**	0.024	1.012–1.177
High	1.130**	0.001	1.048–1.219
Community socioeconomic status			
Low ^a^	–	–	–
Medium	1.001	0.979	0.923–1.085
High	1.025	0.484	0.956–1.099
Community knowledge of modern method			
Low ^a^	–	–	–
Medium	0.927**	0.048	0.861–0.999
High	0.911**	0.014	0.845–0.982
Proportion ever used contraceptive in community			
Low ^a^	–	–	–
Medium	1.021	0.592	0.947–1.101
High	1.086**	0.031	1.007–1.171
Geo-political zone			
North-central ^a^	–	–	–
North-east	0.996	0.929	0.915–1.085
North-west	0.865*	*p* < 0.001	0.798–0.937

^a^RC: *Reference category*, *p < 0.001, **p < 0.05

As community literacy improves from low to medium and high, the likelihood of unmet contraceptive need increased among the women indicating a positive relationship between community literacy level and unmet contraceptive need. Though, the relationship between community socioeconomic status was also positive, but the relationship was without statistical significance. In contrast, as community knowledge of modern contraceptive improved from low to medium and to high, the likelihood of unmet contraceptive need reduced significantly among the respondent. Women who live in communities with high proportion of women who had ever used contraceptive were significantly more likely to have unmet contraceptive need (UOR = 1.086; *p* < 0.05). Place of residence and geo-political zones of the respondents were significantly associated with unmet contraceptive need. Visitation by family planning worker was the only control variable not significantly related to unmet contraceptive need among the women. The variables that showed no statistical significance at the bivariate level, namely, age at first marriage, female autonomy, pregnancy termination, community socioeconomic status, and visitation by family planning worker were excluded from the multivariate analyses.

Table 4 presents the fixed effects of the MLRM. The Wald chi-square tests confirm that the models are adequately fitted. In Model 1, the four maternal characteristics included in the model, namely, maternal age, number of living children, education, and experience of death of a child revealed statistically significant associations with unmet contraceptive need. The inclusion of the community-level characteristics in Model 2 did not cause any change in the statistical significance of the maternal characteristics. In the model, community literacy level, community knowledge of modern contraceptive, and geo-political zones were the community-level characteristics significantly associated with unmet contraceptive need.

**Table 4:** Fixed effects on the likelihood of unmet contraceptive need, Northern Nigeria

Characteristic	Model 1 (Wald chi-sq = 266.1; *p* < 0.001)			Model 2 (Wald chi-sq = 298.1; *p* < 0.001)			Model 3 (Wald chi-sq = 325.2; *p* < 0.001)		
	AOR	*p*-value	95% CI	AOR	*p*-value	95% CI	AOR	*p*-value	95% CI
Maternal age (years)									
15–24 years^a^	–	–	–	–	–	–	–	–	–
25–34 years	0.977	0.622	0.893–1.070	0.964	0.429	0.880–1.056	0.969	0.500	0.885–1.062
35 years or older	0.885	*p* < 0.05	0.792–0.988	0.867	*p* < 0.05	0.776–0.969	0.873	*p* < 0.05	0.780–0.976
Number of living children									
Four or fewer^a^	–	–	–	–	–	–	–	–	–
Five or more	1.801	*p* < 0.01	1.652–1.963	1.808	*p* < 0.01	1.658–1.971	1.813	*p* < 0.01	1.663–1.977
Maternal education									
None^a^	–	–	–	–	–	–	–	–	–
Primary	1.081	0.105	0.984–1.188	1.030	0.548	0.935–1.136	1.013	0.809	0.913–1.123
Secondary	1.124	*p* < 0.05	1.005–1.257	1.044	0.476	0.927–1.175	1.022	0.747	0.896–1.165
Higher	0.869	0.176	0.710–1.065	0.778	*p* < 0.05	0.630–0.960	0.788	*p* < 0.05	0.625–0.993
Death of a child									
Ever experienced^a^	–	–	–	–	–	–	–	–	–
Never experienced	0.882	0.001	0.819–0.949	0.867	*p* < 0.01	0.805–0.934	0.866	*p* < 0.01	0.805–0.933
Community literacy level									
Low^a^				–	–	–	–	–	–
Medium				1.138	0.052	0.999–1.296	1.128	0.069	0.991–1.285
High				1.247	*p* < 0.05	1.054–1.474	1.230	*p* < 0.05	1.041–1.454
Community Knowledge of modern contraceptive									
Low^a^				–	–	–	–	–	–
Medium				0.861	*p* < 0.05	0.766–0.968	0.862	*p* < 0.05	0.767–0.968
High				0.815	0.001	0.721–0.923	0.821	*p* < 0.05	0.726–0.929
Proportion ever used modern contraceptive in community									
Low^a^				–	–	–	–	–	–
Medium				0.989	0.849	0.881–1.110	0.988	0.835	0.880–1.108
High				1.030	0.654	0.906–1.170	1.025	0.700	0.903–1.164
Place of residence									
Urban^a^				–	–	–	–	–	–
Rural				0.909	0.060	0.822–1.004	0.916	0.088	0.829–1.013
Geographic region									
North-central^a^				–	–	–	–	–	–
North-east				0.881	*p* < 0.05	0.788–0.985	0.887	*p* < 0.05	0.793–0.994
North-west				0.827	*p* < 0.01	0.745–0.917	0.832	0.001	0.748–0.926
Barriers to healthcare									
At least one barrier^a^							–	–	–
No barrier							1.162	*p* < 0.05	1.040–1.299
Partner education									
None^a^							–	–	–
Primary							1.016	0.761	0.915–1.128
Secondary							1.100	0.088	0.986–1.228
Higher							1.048	0.514	0.911–1.205
Couple fertility desire									
Both want same^a^							–	–	–
Husband want more							1.057	0.215	0.968–1.153
Husband want fewer							1.011	0.911	0.832–1.229
Don’t know							1.193	*p* < 0.01	1.087–1.309

^a^RC: *Reference Category*, *p* < 0.01 or *p* < 0.05 is statistically significant

In Model 3, four maternal characteristics (maternal age, number of living children, experience of death of a child, and maternal education), and three community characteristics (community literacy level, community knowledge of modern contraceptive, and geo-political zone) were the variables significantly associated with unmet contraceptive need. Women who were 35 years or older were 12.7% less likely to have unmet contraceptive compared to women in the 15–24 years age range (AOR = 0.873; *p* < 0.05, CI: 0.780–0.976). However, women who had five or more living children were 81.3% more likely to have unmet contraceptive need compared to women who had four or fewer number of living children (AOR = 1.813; *p* < 0.001, CI: 1.663–1.977). Women who attained higher education were 21.2% less likely to have unmet contraceptive need compared to uneducated women (AOR = 0.787; *p* < 0.05, CI: 0.625–0.993). Also, women who had never experienced death of a child were 13.4% less likely to have unmet contraceptive need compared to women who had experienced death of a child (AOR = 0.866; *p* < 0.001, CI: 0.805–0.933).

The model further reveals that women who live in communities with high literacy level were 23% more likely to have unmet contraceptive need compared to women in the reference category (AOR = 1.230; *p* < 0.05, CI: 1.041–1.454). On the contrary, women who live in communities with high knowledge of modern contraceptive were 17.9% less likely to have unmet contraceptive need compared to women in communities with low knowledge of modern contraceptive need (AOR = 0.821; *p* < 0.05, CI: 0.726–0.929). Likewise, women in the North-west geo-political zone were 16.8% less likely to have unmet contraceptive need compared to women in the North-central geo-political zone (AOR = 0.832; *p* = 0.001, CI: 0.748–0.926). Barrier to healthcare was the only control variable that reveals significant association with unmet contraceptive need. Table 5 presents random effects of the MLRM. Diagnosis of the model adequacy using the LR test reveals that the empty model as well as the three fitted models were adequate for examining the significance of contextual influence on unmet contraceptive need (*p* < 0.001). However, the results of the ICC only reveal marginal importance of the community level characteristics compared with the maternal characteristics. Across the fitted models, the ICC showed that community factors accounted for less than 5 % of the variation in unmet contraceptive need among the women, which was buttressed by the median odds ratios that indicate a low context effect in the three models.

**Table 5:** Multilevel logistic regression model showing random-effects on unmet contraceptive need, Northern Nigeria

Parameter	Empty Model	Model 1	Model 2	Model 3
Community-level variance (S.E.)	0.139 (0.019)	0.137 (0.019)	0.127 (0.018)	0.123 (0.018)
ICC	4.1%	4.0%	3.7%	3.6%
Log-likelihood	−12,910.7	−12,773.9	−12,756.3	−12,740.8
LR test	χ^2^ = 166.0; *p* < 0.001	χ^2^ = 159.4; *p* < 0.001	χ^2^ = 139.4; *p* < 0.001	χ^2^ = 133.7; *p* < 0.001
Median Odds Ratio (MOR)	0.96	0.96	0.92	0.91

*Notes: p* < 0.001 is statistically significant

## Discussion

This study examined the extent to which maternal and community factors were associated with unmet contraceptive need in Northern Nigeria. The study not only provided additional information on the prevalence and associated factors of unmet contraceptive need, but has also made two key significant contributions to the literature on unmet need for family planning. Firstly, the study focused on Northern Nigeria which is the region with the poorest contraceptive prevalence in the country, and has only been covered in few studies on unmet contraceptive need in the region [20–22]. The situation of unmet need for family planning in Northern Nigeria has therefore been brought further to the fore of national family planning awareness, which may draw the attention of family planning programming officers in the country. Secondly, the study extended search for significant correlates of unmet contraceptive need in Nigeria by examining the likely community characteristics that may shape the level of unmet need for family planning in Northern Nigeria. This addressed the limitation of previous studies in the region [20–22], and provides information on wider range of variables to be targeted by family planning policy makers in the region. Based on the findings five key issues emerged from the study.

One, the prevalence of unmet contraceptive need in Northern Nigeria is 18%. This prevalence is higher than the 10.3% reported in an earlier study in the region [21] and much higher than the 2% reported in a recent study in the region [22]. This variation may be explained by the fact that the previous studies in the region [20–22] were small scale surveys compared to the NDHSs which were nationally representative and covered more women in the region. The result is however similar to the 17.1% reported in a study in Indonesia [15], the 17.4% reported by a study in Ethiopia [16], and the 17.4% reported in a study conducted in Iran [14]. However, the level of unmet contraceptive need found in this study is much lower than the 31.1% reported by a study in North-west region of Cameroon [18], the 44% found in a study in Dessie Town of Ethiopia [17], as well as the 32.4% reported in a study conducted in Burundi [19]. The level of unmet contraceptive need found in this study is thus comparable to findings elsewhere, and suggests need for fresh initiatives in Northern Nigeria to reduce unmet need for family planning among childbearing women in the region.

This is particularly important because high unmet contraceptive need further expose women to unintended pregnancies, unsafe abortion, and hinder women from effective economic participation, which also have implications for family health [6–9]. Bearing in mind, that Northern Nigeria is a high fertility zone in the country [29], there is need for urgent steps to be taken to reduce unmet need for family planning in Northern Nigeria. This may include expansion of existing family planning delivery points to cover all communities including rural and remote areas in the region. Such initiative should take advantage of the existing negative relationship between community knowledge of modern contraceptive and unmet contraceptive need as found in the study. In communities with high knowledge of modern contraceptive, the odds of unmet need were lower to indicate that expansion in knowledge of modern contraceptive may further reduce unmet need among women. It is therefore important that expansion in service delivery points should be accompanied with expansion in women’s education which is currently poor as found in this study, as this may boost awareness and utilisation of modern contraceptives among women in the region.

Two, the study found that women who had five or more living children had higher likelihood of unmet contraceptive need in Northern Nigeria. This situation is detrimental to maternal and child health in the region due to widespread evidence that high parity among women elevates the risk of adverse maternal and child health outcomes [32–36]. It is therefore important for additional contraceptive information, education and communication programme in the country to specifically target women with high parity in Northern Nigeria. Such programme could be designed and implemented by Primary Health Care agencies which are the nearest health facility to most women in the region.

Three, the study reveal that women with higher education had the least likelihood of unmet contraceptive need in Northern Nigeria which was consistent with findings in previous Nigerian studies [11, 12]. This implies that initiatives to improve women’s access to formal education in the region should not be limited to primary and secondary educational levels but should seeks to make higher education more accessible to women in the region. Higher education for women not only provides them with economic empowerment, it also enhances their ability to take reproductive decisions with little male involvement. Though, higher education among women may also create some problems such as the problems associated with delayed marriage and childbearing, but such problem can be properly handled through adequate family life education curriculum. In spite of the significance of maternal education, it was revealed in the study that high community literacy level was associated with higher odds of unmet contraceptive need. This seems counter-productive but may be explained by the fact that when there is high proportion of women who had secondary or higher education in the community, there is also higher likelihood of postponement of marriage or childbearing among women in the community. In such community, women will most likely use contraceptive for spacing and not limiting. This may create unmet contraceptive need among women particularly when family planning delivery is poor in the community.

Four, the study reveals that women who had never experienced death of a child had less likelihood of unmet contraceptive need. This finding has two implications. Firstly, it may suggest that women who had lost a child in the region tend to desire replacement of the dead child irrespective of current number of living children. For women still at the lower echelons of the reproductive life span, such fertility desire may not be injurious to their reproductive health, but for women already in advanced reproductive age who may no longer have fertility desire such attempt to replace a dead child may lead to serious health complications. Secondly, it tends to draw attention to boosting child survival in the region. Presently, child health is poorest in Northern Nigeria compared to Southern Nigeria [38] and needs to be improved upon to discourage some women from continuing to have deliveries with the mind-set that some of the new-borns may not survive to adulthood, hence another pregnancy is desired as possible replacement.

Five, the study revealed that the odds of unmet contraceptive need was higher among women who had no barrier to accessing healthcare. Ordinarily, women who had no barrier to accessing healthcare should have reduced unmet contraceptive need since such women will have more likelihood of receiving facility-based counselling and education on family planning. The results however may also indicate that in some situations, having access to healthcare services may not necessarily translate to utilisation of the services. This is possible in many Islamic settings such as Northern Nigeria where women may not utilise available services due to preference for female healthcare providers or personnel who may not be readily available in some health facility, particularly in rural and remote areas. Thus, repositioning family planning services in Northern Nigeria may require the development and implementation of more religion or culture specific strategies. This may be particularly more relevant in the North-east and North-west geopolitical zones of the region because the two zones have the poorest levels of contraceptive prevalence in the country.

The analysis performed in the study suffers from two drawbacks. One, the study pooled data from surveys conducted in 2008 and 2013. This will treat the sample analysed as sampling done with replacement, and will not allow the findings to be specifically linked to either to 2008 or 2013 but jointly as a single period of 2008–2013. It is therefore important to understand the inference made in the study with caution despite the precision attributable to pooled data. Also, the cross-sectional nature of the data did not allow the investigation of causality. Hence, the analyses were limited to investigating associations between the research variables.

## Conclusion

This study analysed maternal and community factors associated with unmet contraceptive need in Northern Nigeria. Data were extracted from the 2008–2013 Nigeria Demographic and Health Surveys. Findings revealed that some maternal and community factors were significantly associated with unmet contraceptive need in Northern Nigeria. However, based on the results of the ICC, individual maternal characteristics showed more significance than community characteristics. The expansion of existing family planning delivery points to cover all communities including rural and remote areas in the region is imperative.

## Data Availability

The dataset supporting the conclusions of this article is available online at https://dhsprogram.com/data/

## Abbreviations

DHS: Demographic and Health Survey
MLRM: Multilevel Logistic Regression Model
NDHS: Nigeria Demographic and Health Survey
